# Interval Training in Sports Medicine: Current Thoughts on an Old Idea

**DOI:** 10.3390/jcm11185468

**Published:** 2022-09-17

**Authors:** Sascha Ketelhut, Reinhard G. Ketelhut, Burkhard Weisser, Claudio R. Nigg

**Affiliations:** 1 Institute of Sport Science, University of Bern, 3012 Bern, Switzerland; 2 Cardiology and Sports Medicine, Medical Center Berlin (MCB), 10559 Berlin, Germany; 3 Institute of Sports Science, Christian-Albrechts-University of Kiel, 24118 Kiel, Germany

## 1. Introduction

In light of the global physical inactivity pandemic, the increasing prevalence of non-committable diseases, and mounting healthcare costs, effective and feasible prevention and treatment approaches are urgently needed. In this regard, the value of physical activity and exercise are well recognized [1,2]. An optimal dose, frequency, duration, intensity, and type of exercise can improve aerobic and metabolic capacity and cardiac function, reduce the risks of cardiovascular disease, diabetes, hypertension, and some cancers and prevent musculoskeletal disorders and mental illness [3,4].

For many years, exercise training has been recommended as an integral part of prevention and rehabilitation approaches by major international societies [5,6]. Most traditionally, exercise guidelines recommend moderate-intensity continuous training (MICT) at an intensity of 60–85% of maximum heart rate [7,8,9]. Based on broad evidence from randomized controlled trials, MICT has become a widely recommended exercise protocol. Nonetheless, long-term adherence remains challenging for most people, with a lack of time being cited as one of the most common barriers [10].

In the last two decades, there has been a resurgence in interest in interval training. Interval training refers to an intermittent style of exercise in which repeated bouts of exercise are broken up by active or passive recovery periods. Research indicates that similar, if not greater, improvements in VO_2_max and other performance and health variables are possible with interval training compared to continuous aerobic training [11,12]. The advantage of intermittent exercise is that it enables individuals to achieve greater total exercise time at higher exercise intensities compared to continuous training. It is widely accepted that exercise of higher intensity leads to greater adaptations and more pronounced health benefits [13].

One type of interval training that has progressively increased in popularity among scholars and practitioners is high-intensity interval training (HIIT). The latest exercise guidelines suggest HIIT as an alternative protocol to improve aerobic capacity and cardiac function [14]. Despite the promising effects of intermittent exercise programs, there are still numerous open questions concerning the program designs, the effects for specific target groups, and how these programs can be made suitable for everyday use.

## 2. Definition and Protocols

Starting as a training approach for athletes, interval training has advanced as a training modality applied in prevention and treatment programs for different diseases and health issues. Lately, research into the physiological adaptations to interval training in healthy individuals and people with diseases has exploded. Today, HIIT is recommended in particular as an adjunct to MICT in the treatment guidelines of various professional societies. However, interval training can also involve less demanding activity characterized by alternating periods of light and moderate exercise (e.g., interval walking).

Notably, the term HIIT is not consistently defined, and multiple descriptions and exercise protocols are used [15]. In the current literature, a range of protocols are employed, which consist of exercise bouts lasting a few seconds and intensities close to maximum [16] and bouts lasting up to 4 min and correspondingly lower intensities [17,18]. The differences in the training variables are expected to reflect changes in the metabolism and adaptations of organic systems [19]. 

A distinctive feature of intermittent exercise training is the possibility to manipulate the different training variables, including the duration of the bouts and recovery phases, the intensity of bouts and recovery, and the total amount of bouts/recovery. This leads to numerous different protocols, making this training approach infinitely variable and individually adjustable.

## 3. Efficacy

There is growing interest in interval training, especially HIIT, due to the relatively robust evidence of its positive effects on cardiovascular and metabolic function in both healthy and diseased populations [11,20]. Multiple meta-analyses have reported superior effectiveness of HIIT compared with traditional MICT for improving cardiorespiratory fitness [21,22,23]. As accumulated evidence suggests that aerobic capacity (VO_2_max) is one of the strongest predictors of all-cause mortality [24], it has become a major treatment goal to improve VO_2_max in patients with lifestyle-related diseases [25].

Apart from cardiorespiratory fitness, HIIT shows better improvements in risk factors, vasculature [26], respiration [27], autonomic function [28], cardiac function [16,27], inflammation [16], quality of life [29], and endothelial function than MICT [25].

### Metabolic Effects

In addition to cardiovascular effects, physical exercise has been shown to improve metabolism on various levels. For an increase in aerobic oxidative capacity, traditionally, only MICT was recommended. In contrast, HIIT is now perceived as a means to increase maximal anaerobic capacity and not as a typical training modality to improve lipid oxidation and aerobic carbohydrate metabolism. In a landmark study, Burgomaster et al. [30] compared the influence of sprint interval training (SIT) with a classical MICT. SIT is basically a form of HIIT, consisting of four to six repetitions of 30 s “all out” periods. Endurance training consists of 40–60 min cycling 5 days per week at approximately 65% of VO_2_ peak.

Not only was the increase in exercise capacity between the two groups comparable, but there was also no difference in the effect on intracellular enzymes for lipid and carbohydrate oxidation. This and other studies have shown that despite markedly shorter training times, similar changes in lipid oxidation and glycogen utilization can be obtained by HIIT [30].

## 4. Feasibility and Safety

A benefit of HIIT is that it is relatively time efficient compared with MICT. Furthermore, many individuals report greater enjoyment during HIIT and show at least similar overall training adherence compared with MICT [31]. Thus, HIIT has been proposed as a time-efficient alternative or adjunct exercise approach to traditional MICT [11,12].

However, adherence to physical activity and exercise programs is known to quickly drop after completion of a supervised training period, and with it exercise-mediated benefits [32]. Even though HIIT sessions are relatively short, they require an extremely high level of subject motivation [18]. Given the extreme nature of HIIT, there is reasonable doubt that the general population could adopt this training mode over an extended period. This is supported by [33], who reported a high dropout rate following the supervised exercise period. Apart from dropout, adherence to the prescribed exercise intensity presents a major problem, especially in untrained populations [34,35].

Another highly relevant topic concerning interval training and HIIT is safety. Participant safety is central to using HIIT as a tool to reduce the risk of cardiometabolic disease among adults, especially those with cardiometabolic risk factors, diagnosed cardiovascular diseases, or other chronic diseases.

According to a systematic review by [36], the incidence of adverse events during or 24 h after a single HIIT session in patients with cardiometabolic diseases was around 8%. A review by Rognmo et al. [37] addressed the risk of cardiovascular events during HIIT and MICT in a total of 175,820 training hours. The authors reported one fatal cardiac arrest during MICT (129,456 exercise hours) and two nonfatal cardiac arrests during HIIT (46,364 exercise hours). An even lower incidence of adverse cardiovascular events was reported by Wewege et al. [38]. The authors identified only one major (and nonfatal) cardiovascular adverse event per 11,333 training hours in 23 studies involving 547 participants. Thus, it can be concluded that the risk of a cardiovascular event is low after HIIT.

## 5. Open Research Questions

The topic of interval training in sports science has attracted considerable interest among scholars and practitioners. This level of attention has coincided with a large number of related publications over the last few years. Future research should aim to further advance our understanding of the effect of interval training on indices of health in different populations and settings. Therefore, we summarize the future directions to motivate researchers in the field of sports medicine.

### 5.1. Determine Appropriate Protocol Recommendations for Different Patient Populations and Target Groups

Even though studies have assessed the effects of HIIT in different target groups, there is still insufficient evidence available to determine how the effects are influenced by age, sex, race/ethnicity, or socioeconomic status. There is still a paucity of research addressing interval training in younger demographics, and specific diseased populations. Additionally, a considerable gender gap can be identified in the current body of literature, with the number of studies addressing women being much lower. 

### 5.2. Compare and Systematically Review/Meta-Analyze the Effects of Different Types of HIIT-Related Programs

As stated earlier, a distinctive feature of intermittent exercise training is the possibility to manipulate the different training variables making this training approach infinitely variable. To date, it is unclear which approach is the most effective for which target group, setting, and outcome. Apart from efficacy, program designs should aim for higher adherence. The optimal exercise prescription is likely that which can be maintained long-term [39]. Therefore, the merit of exercise approaches must be evaluated not solely based on health-related benefits that they produce in the short term but on their potential for long-term maintenance. The question should be which program shows the greatest effectiveness at the optimum cost.

### 5.3. Conduct Long-Term Randomized Controlled Trials Focusing on Medical Endpoints

A review of previous the literature on interval training reveals that most interventions lasted only a few months and long-term studies are lacking. Even though significant training effects are possible within a short period of time, it is unclear how stable the upregulation of various parameters is. Long-term studies report that a change in VO_2_max was predicted by longer HIIT intervention duration but not by total time performing HIIT [20]. Furthermore, studies focusing on relevant medical endpoints are highly warranted, especially as research addressing the effects of HIIT on mortality is still sparse. 

### 5.4. Capitalizing on Technology and Digital Approaches to Increase Adherence and Reduce Supervision 

The digitalization and increasing reliance on technology presents unprecedented opportunities in reach, interactivity, tailoring, immediacy, and connections in order to promote, disseminate, monitor, and supervise exercise programs. 

Smartphone applications, exergames (combination of video games and exercise), and wearables can especially help to promote, monitor, and supervise interval training programs not only in prevention and rehabilitation settings but, moreover, in home-based intervention approaches. Further research on the feasibility and efficacy of such approaches is highly warranted.

## Data Availability

Not applicable.
